# The Role of Notch3 Signaling in Cancer Stemness and Chemoresistance: Molecular Mechanisms and Targeting Strategies

**DOI:** 10.3389/fmolb.2021.694141

**Published:** 2021-06-14

**Authors:** Mengxi Xiu, Yongbo Wang, Baoli Li, Xifeng Wang, Fan Xiao, Shoulin Chen, Lieliang Zhang, Bin Zhou, Fuzhou Hua

**Affiliations:** ^1^ Department of Anesthesiology, The Second Affiliated Hospital of Nanchang University, Nanchang, China; ^2^ Key Laboratory of Anesthesiology of Jiangxi Province, Nanchang, China; ^3^ Department of Gastroenterology, The First Affiliated Hospital of Nanchang University, Nanchang, China

**Keywords:** cancer, Notch3, mechanism, targeted therapy, cancer biology

## Abstract

Aberrant Notch signaling profoundly affects cancer progression. Especially the Notch3 receptor was found to be dysregulated in cancer, where its expression is correlated with worse clinicopathological features and poor prognosis. The activation of Notch3 signaling is closely related to the activation of cancer stem cells (CSCs), a small subpopulation in cancer that is responsible for cancer progression. In addition, Notch3 signaling also contributes to tumor chemoresistance against several drugs, including doxorubicin, platinum, taxane, epidermal growth factor receptor (EGFR)–tyrosine kinase inhibitors (TKIs) and gemcitabine, through complex mechanisms. In this review, we mainly focus on discussing the molecular mechanisms by which Notch3 modulates cancer stemness and chemoresistance, as well as other cancer behaviors including metastasis and angiogenesis. What’s more, we propose potential treatment strategies to block Notch3 signaling, such as non-coding RNAs, antibodies and antibody-drug conjugates, providing a comprehensive reference for research on precise targeted cancer therapy.

## Introduction

**FIGURE 1:**
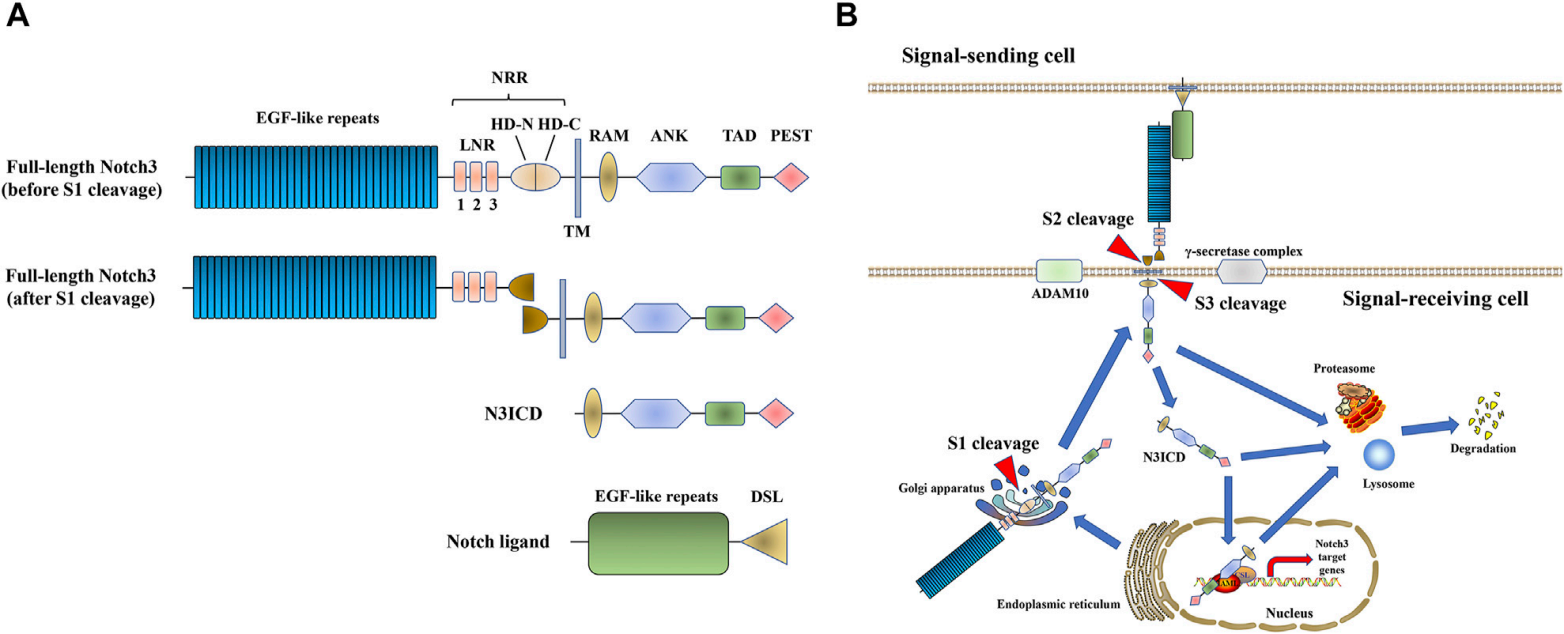
Notch3 signaling. **(A)** The structure of Notch3 and its ligand. The full-length Notch3 protein consists of 34 epidermal growth factor (EGF) repeats, a negative regulatory region (NRR) [composed of three Lin-Notch repeats (LNRs) and a heterodimerization domain (HD)], a transmembrane domain, an RBPJ-association module (RAM) domain, seven ankyrin (ANK) repeats, two nuclear localization signals (NLS), a transactivation domain (TAD) and a C-terminal domain rich in proline, glutamic acid, serine, and threonine (PEST domain) (Xiu and Liu, 2019). The Notch ligand consists of EGF repeats and a Delta/Serrate/LAG-2 (DSL) domain. **(B)** Notch3 signal transduction process.

Notch signaling is a highly conserved among multicellular organisms, and it is involved in cell fate decision, cell proliferation/differentiation, as well as cell lineage specification (Bray, 2006; Bray, 2016). The activation of Notch signaling is mediated by cell-to-cell interactions with a Notch ligand. In mammals, there are four Notch receptors (Notch1-4) and five ligands [Jagged (JAG)1, 2 and Delta-like ligand (DLL)1, 3 and 4]. Before it is trafficked to the cell membrane, the full-length Notch receptor undergoes initial cleavage, also called S1 cleavage, in the Golgi apparatus. When a Notch ligand (JAG or DLL) in the cell membrane of an adjacent signal-sending cell interacts with a Notch receptor in the cell membrane of the signal-receiving cell, the Notch receptor is activated and undergoes another proteolytic cleavage. This so-called S2 and S3 cleavage steps are induced by A Disintegrin And Metalloprotease domain 10 (ADAM10) and the γ-secretase complex, respectively (Bray, 2006; Kopan and Ilagan, 2009; Bray, 2016). Subsequently, the Notch intracellular domain (NICD) is released and translocated into the nucleus, where it binds to the effector DNA-binding transcription factor CSL. The latter then recruits the transcription co-activator mastermind-like protein (MAML) to induce the transcription of downstream target genes. Finally, the Notch receptor or NICD undergoes proteasomal/lysosome degradation (Bray, 2006; Kopan and Ilagan, 2009; Bray, 2016; Xiu and Liu, 2019) (Figure 1, shown on the example of Notch3 signaling).

**FIGURE 2:**
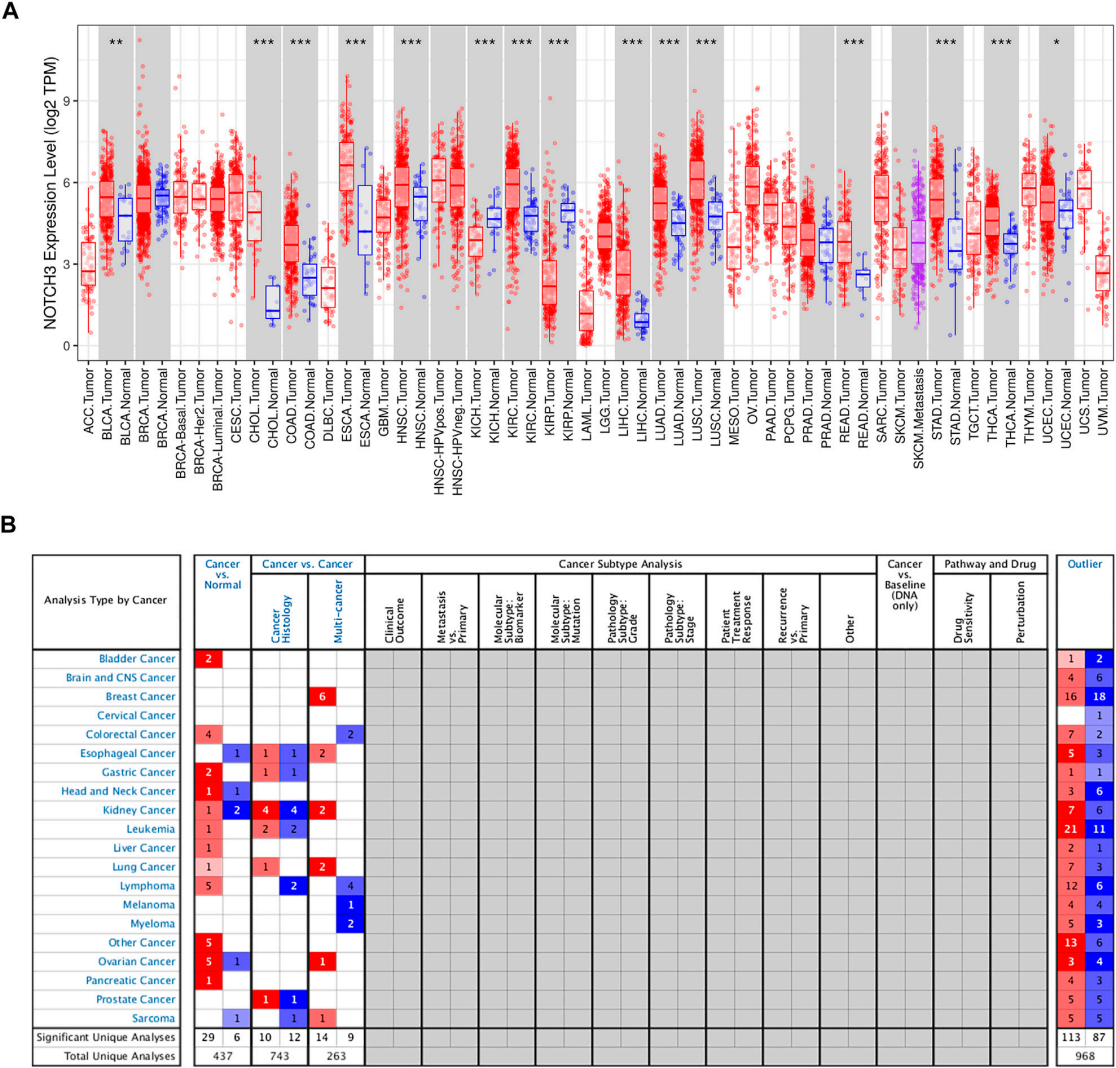
The expression of Notch3 in human cancers. **(A)** The Notch3 expression levels of different cancer types in TCGA were determined using TIMER (https://cistrome.shinyapps.io/timer/) (*p < 0.05, **p < 0.01, ***p < 0.001). **(B)**. Notch3 expression levels in different cancer types from the Oncomine database (www.oncomine.org/).

The Notch3 receptor is encoded on chromosome 19p13.12 (19: 15159038-15200995), spanning 33 exons (https://www.ncbi.nlm.nih.gov/gene/4854). The aberrant high expression of Notch3 is common in human cancer tissues, as shown in several studies (Giovannini et al., 2009; Park et al., 2010; Zhang et al., 2011; Rahman et al., 2012; Hu et al., 2013; Ye et al., 2013; Ozawa et al., 2014; Liu et al., 2016b; Zhang et al., 2017; Tang et al., 2019; Xu et al., 2019), as well as the Cancer Genome Atlas (TCGA) and Oncomine database (Figure 2). High Notch3 expression in cancer tissues is correlated with a series of clinicopathological features, such as large tumor size, advanced TNM stage, high pathological grade and tumor metastasis, as well as a diminished prognosis of cancer patients, such as poor overall survival (OS), disease-free survival (DFS), relapse-free survival (RFS) and progression-free survival (PFS) (Table 1) (Park et al., 2010; Zhang et al., 2011; Mann et al., 2012; Rahman et al., 2012; Alqudah et al., 2013; Hu et al., 2013; Ye et al., 2013; Zhou et al., 2013; Ozawa et al., 2014; Yuan et al., 2015; Liu et al., 2016a; Liu et al., 2016b; Ma et al., 2016; Zhou et al., 2016; Kim et al., 2017a; Xue et al., 2017; Yu et al., 2017; Zhang et al., 2017; Lin et al., 2018; Tang et al., 2019; Xu et al., 2019; Zhang et al., 2019).

**TABLE 1:** The clinical significance of Notch3 in different types of cancer.

Cancer type	Sample count	Clinicopathological and prognostic significance of high Notch3 expression in cancer	References
Lung adenocarcinoma	20	Predicts poor OS	Zhang et al. (2019)
Non-small-cell lung carcinoma	104	Predicts poor OS and DFS	Ma et al. (2016)
	3663 (Meta-analysis)	Predicts poor OS	Yuan et al. (2015)
	131	Predicts poor OS	Ye et al. (2013)
		Correlated with advanced TNM stage and lymph node metastasis	
Hepatocellular carcinoma	86	Predicts poor OS	Zhou et al. (2013)
		Correlated with metastasis, venous invasion and satellite lesions	
	95	Predicts poor OS	Hu et al. (2013)
		Correlated with large tumor size, multiple tumors and advanced TNM stage	
Hepatitis B virus-related hepatocellular carcinoma	465	Predicts poor OS and RFS	Yu et al. (2017)
Colorectal carcinoma	305	Predicts poor relapse-free survival and distant metastasis	Ozawa et al. (2014)
		Correlated with low differentiation degree and venous invasion	
Breast carcinoma	72	Correlated with positive expression of ERα and PR, with reduced lymph node metastasis	Lin et al. (2018)
Triple-negative breast carcinoma	105	Correlated with advanced TNM stage and lymph node metastasis	Xue et al. (2017)
Tongue carcinoma	74	Correlated with advanced TNM stage	Zhang et al. (2011)
Pancreatic adenocarcinoma	42	Predicts poor OS and DFS	Mann et al. (2012)
		Correlated with lymph node metastasis	
	101	Predicts poor OS	Zhou et al. (2016)
		Correlated with advanced TNM stage, high pathological grade, lymph node metastasis and venous invasion	
Gliomas	60	Predicts poor OS	Alqudah et al. (2013)
Ovarian carcinoma	42	Predicts poor OS and PFS	Park et al. (2010)
	61	Predicts poor OS and PFS	Rahman et al. (2012)
	86	Predicts poor OS	Kim et al. (2017a)
		Correlated with advanced TNM stage, lymph node metastasis, distant metastasis, and chemoresistance	
	120	Predicts poor OS	Liu et al. (2016b)
		Correlated with advanced TNM stage, high pathological grade, advanced histological type, lymph node metastasis, and ascites	
	266	Predicts poor OS and PFS	Xu et al. (2019)
Urothelial carcinoma	59	Predicts poor OS	Zhang et al. (2017)
		Correlated with distant metastasis	
Gallbladder carcinoma	126	Predicts poor OS	Liu et al. (2016a)
		Correlated with large tumor size, advanced TNM stage, invasion, lymph node metastasis, and inability of surgical resection	
Osteosarcoma	70	Predicts poor OS	Tang et al. (2019)
		Correlated with tumor metastasis	

Notes: OS: overall survival; DFS: disease-free survival; TNM: tumor node metastasis; RFS: relapse-free survival; ERα: estrogen receptor α; PR: progesterone receptor; PFS: progression-free survival.

**FIGURE 3:**
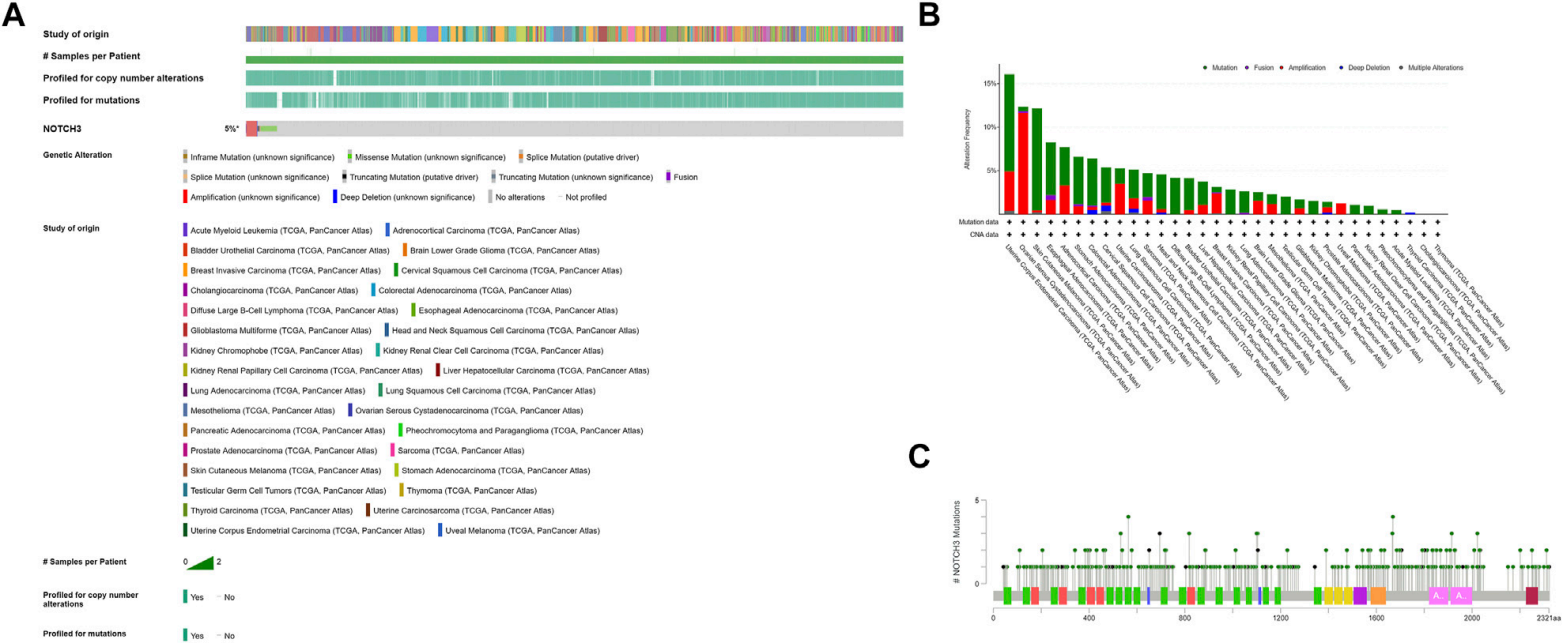
Alterations of Notch3 gene in human cancers from TCGA were determined using the cBio Cancer Genomics Portal (http://cbioportal.org). **(A)** Types of Notch3 gene alterations found in cancer. **(B)** Cancer-specific Notch3 gene alterations. **(C)** Cancer-related mutations of Notch3, including 354 missense mutations (green dots) and 55 truncation mutations (black dots).

Notch3 overexpression in cancer is mainly caused by alterations of the Notch3 gene. According to TCGA, the Notch3 gene was altered in 5% of cancer samples, mainly via amplification and mutation (Figure 3). Notch3 has been reported to be amplified in 10–25% of ovarian carcinoma (OC) (Park et al., 2006; Etemadmoghadam et al., 2009; Cancer Genome Atlas Research Network, 2011; Hu et al., 2014). Among all cancer types in the TCGA database, OC has the highest Notch3 amplification rate (11.64%, 68 of 584 cases) (Figure 3). In addition to amplification, mutations in the negative regulatory region (NRR) and proline (P), glutamic acid (E), serine (S), threonine (T)-rich (PEST) domains of Notch3 gene can cause Notch3 activation (gain-of-function/activating mutations), as seen in human T-cell acute lymphoblastic leukemia (T-ALL) (Bernasconi-Elias et al., 2016).

Notch signaling plays complex roles in regulating cellular behaviors during cancer progression, and each Notch receptor has its specific pattern (Majumder et al., 2021). A major role of Notch3 is maintaining the stemness of cancer stem cells (CSCs). As a population of self-renewing cells with high tumorigenic potency, CSCs are found to be activated by Notch3 signaling in several kinds of cancer and contribute to cancer progression through complex mechanisms (See *Notch3 and Cancer Stem Cell Properties*). Another main feature of Notch3 signaling is to induce tumor resistance against several kinds of chemotherapeutic drugs, including doxorubicin, platinum, taxane, epidermal growth factor receptor (EGFR)–tyrosine kinase inhibitors (TKIs) and gemcitabine (See in *Notch3 and Drug Resistance*). Of note, Notch3-supported CSC activity is also involved in the mechanisms of tumor chemoresistance, as well as tumor metastasis and angiogenesis, indicating the key role of Notch3 signaling in cancer (Sullivan et al., 2010; Xiao et al., 2011; McAuliffe et al., 2012; Cheung et al., 2016a; Sansone et al., 2016; Kim et al., 2017a; Kim et al., 2017b; Jeong et al., 2017; Wang et al., 2018a; Leontovich et al., 2018; Liu et al., 2018; Papadakos et al., 2019; Fan et al., 2020; Fang et al., 2020; Mansour et al., 2020).

There are numerous published review articles on the effects of Notch signaling in cancer treatment (Giovannini et al., 2016; Bellavia et al., 2018; Giuli et al., 2019; Katoh and Katoh, 2020). By contrast, this review mainly focuses on the underlying Notch3-related molecular mechanisms that regulate cancer stemness and chemoresistance. In addition, the relationships between Notch3 and other tumor biological characteristics, including metastasis and angiogenesis are also discussed. Finally, we summarize known Notch3-targeting strategies/methods for cancer therapy. Overall, this review provides comprehensive information on the role of Notch3 signaling in cancer and its value as a therapeutic target.

## Notch3 and Cancer Stem Cell Properties

**FIGURE 4:**
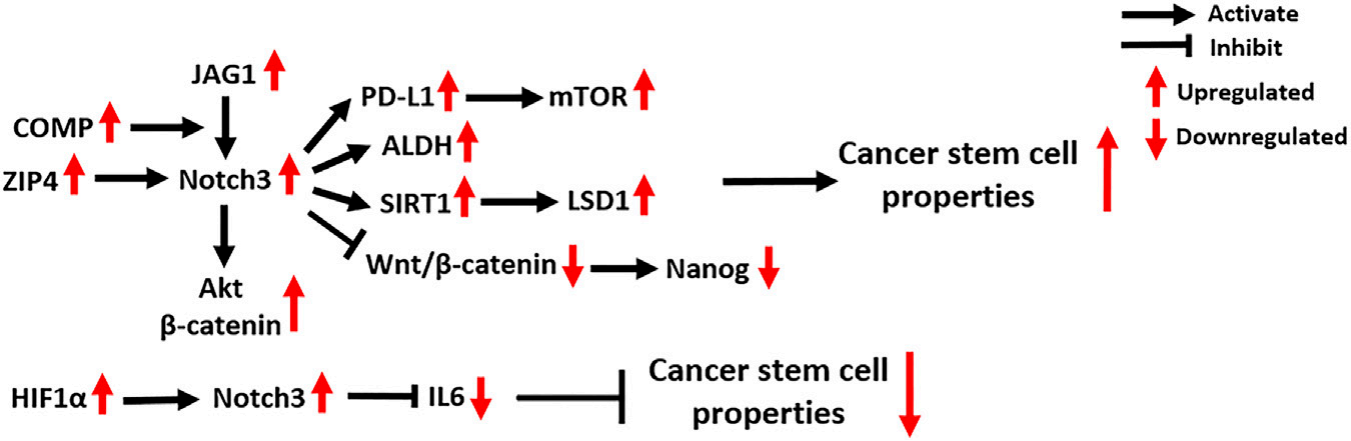
Notch3-related molecular mechanisms in cancer stem cells. COMP: Cartilage Oligomeric Matrix Protein; Akt: AKT Serine/Threonine Kinase; PD-L1: Programmed Death Ligand 1; ALDH: Aldehyde dehydrogenase; SIRT1: Sirtuin 1; LSD1: Lysine-specific demethylase 1; HIF1α: Hypoxia Inducible Factor 1 Subunit Alpha; IL6: Interleukin 6.

Tumor initiation and progression is driven by a small population of cancer cells with self-renewal and tumor-formation capacity, known as CSCs (Dawood et al., 2014). The activation of Notch3 signaling is widely found in CSCs, where it regulates their abundance and activity through several molecular mechanisms (Figure 4). The expression of aldehyde dehydrogenase (ALDH), a recognized CSC marker, is significantly positively correlated with Notch3 expression, as seen in OC, lung carcinoma (LC), hepatocellular carcinoma (HCC) and breast carcinoma (BC) (Sullivan et al., 2010; Xiao et al., 2011; Zhang et al., 2015; Kim et al., 2017a). Suppression of Notch3 signaling in LC cells by treatment with either a γ-secretase inhibitor (GSI) or short hairpin RNA (shRNA) against Notch3 resulted in a significant decrease of ALDH^+^ CSCs, indicating that Notch3 is critical for ALDH expression (Sullivan et al., 2010).

In OC, the zinc transporter ZIP4 was identified as a novel CSC marker that physically interacts with Notch3 and activates Notch3 signaling (Fan et al., 2020). Several studies also found that the activation of Notch3 signaling enhances CSC activity, especially in chemoresistant OC tumors (Kim et al., 2017b; Jeong et al., 2017; Fang et al., 2020), and the relevant mechanisms are discussed in *Platinum and Taxane*.

In HCC, the activation of Notch3 signaling was found to inhibit Wnt/β-catenin signaling and increase the expression of the stemness-related protein Nanog, which promotes the maintenance of the CSC population, thereby contributing to the pathogenesis of HCC (Zhang et al., 2015). In addition, Notch3 signaling in liver CSCs is supported by cancer-associated fibroblasts in the tumor microenvironment and maintains tumor cell self-renewal (Liu et al., 2018). Mechanistically, Notch3 signaling activates LSD1, a histone-modifying enzyme that promotes cancer stemness, by inducing its deacetylation by activating the class-III histone deacetylase (HDAC) SIRT1 (Liu et al., 2018).

In the tumor microenvironment of BC, a secreted protein named Cartilage Oligomeric Matrix Protein (COMP) physically bridges Notch3 and JAG1 on the cell membrane of CSCs, thus driving JAG1/Notch3 signaling and subsequently activating the β-catenin and Akt signaling pathways to maintain CSC status (Papadakos et al., 2019). A recent study showed that Notch3 signaling contributes to the overexpression of the T-cell inhibitory molecule PD-L1 in breast CSCs by activating mTOR signaling (Mansour et al., 2020). Specific knockdown of Notch3 can downregulate PD-L1 expression on CSCs and reduce CSC activity, providing a novel strategy for anti-PD-L1 combination therapies (Mansour et al., 2020). In addition to the mechanisms that promote CSC activity, Notch3 signaling is also found to reduce the population of breast CSCs by negatively regulating IL6 (Wang et al., 2018a). Furthermore, the activation of HIF1α in response to hypoxia is involved in Notch3-mediated IL6 inhibition in breast CSCs via direct binding to the Notch3 promoter. The combination of Notch and IL6 inhibitors significantly decreases the abundance of breast CSCs and inhibits BC growth, suggesting it might serve as a novel therapeutic strategy for treating Notch3-expressing BC (Wang et al., 2018a).

## Notch3 and Drug Resistance

**FIGURE 5:**
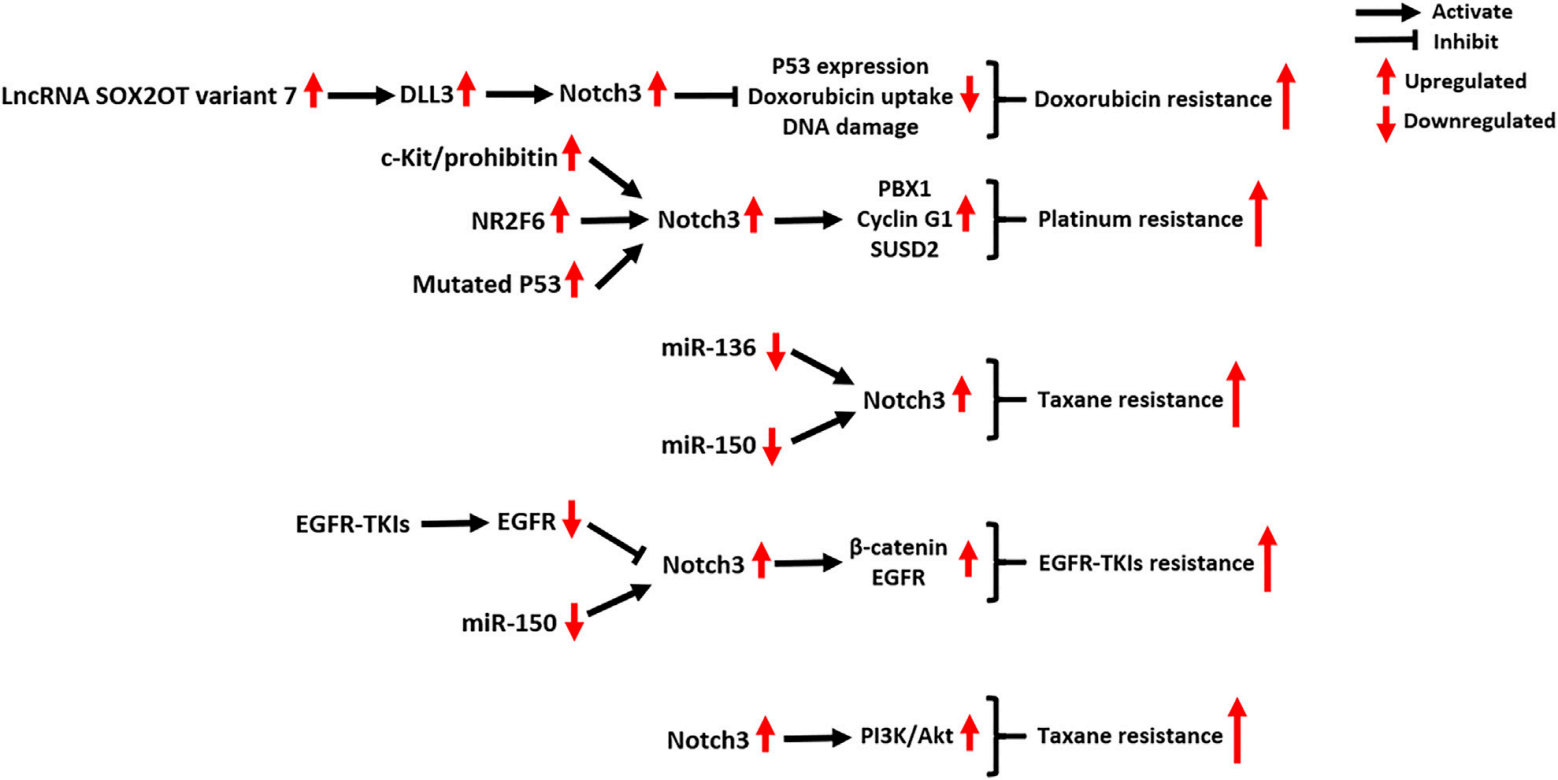
Notch3-related molecular mechanisms in cancer drug resistance. NR2F6: Nuclear Receptor Subfamily 2 Group F Member 6; PBX1: PBX Homeobox 1; SUSD2: Sushi Domain Containing two; EGFR-TKIs: epidermal growth factor receptor–tyrosine kinase inhibitors; PI3K: phosphatidylinositol 3-kinase; Akt: AKT Serine/Threonine Kinase.

A large number of studies have shown that Notch3 signaling is closely related to the ability of tumors to chemotherapy. Here, we mainly introduce the roles of Notch3 in the resistance of tumors to five kinds of chemotherapeutic drugs (doxorubicin, platinum, taxane, EGFR-TKIs and gemcitabine), whose mechanisms are comparatively well-understood (Figure 5).

### Doxorubicin

The resistance of tumor cells to doxorubicin, a DNA topoisomerase II inhibitor, is associated with the activation of Notch3 signaling (Giovannini et al., 2009; Michishita et al., 2011; Wang et al., 2018b). In HCC cells, Notch3 signaling contributes to doxorubicin resistance by inhibiting p53 expression, doxorubicin uptake and DNA damage, which can be reversed by Notch3 depletion (Giovannini et al., 2009). In osteosarcoma, both *in vivo* and *in vitro* experiments indicate that the lncRNA SOX2OT variant seven can activate DLL3/Notch3 signaling, maintaining the stemness and doxorubicin-resistance of tumor cells (Wang et al., 2018b). Treatment of osteosarcoma cells with epigallocatechin gallate, a polyphenol from green tea, can counteract the SOX2OT-7/DLL3/Notch3 axis, thus inhibiting cancer progression (Wang et al., 2018b).

### Platinum and Taxane

Notch3 signaling plays a critical role in tumor resistance to platinum, taxane, or their combination chemotherapy regimens, especially in OC. In a clinical study of 61 OC patients, the high expression of Notch3 was correlated with shorter PFS and OS in patients with stage III and IV disease treated with a standard platinum and taxane chemotherapy regimen (Rahman et al., 2012). *In vitro* experiments in OC cell lines also confirmed that Notch3 was significantly overexpressed in cisplatin-resistant A2780cis cells (2.5-fold) and paclitaxel (PTX)-resistant SKpac cells (25.5-fold) compared to chemo-sensitive A2780 cells (Kim et al., 2017a).

In cisplatin-resistant OC, Notch3 signaling was found to be induced by the activation of the c-Kit/prohibitin axis and the nuclear orphan receptor NR2F6, as well as P53 mutations (Li et al., 2019; Xu et al., 2019; Fang et al., 2020). The activation of Notch3 signaling activates the stem cell reprogramming factor PBX1, which supports the activity of CSCs contributing to platinum chemoresistance (Fang et al., 2020). In addition, Notch3 signaling also upregulates the expression of Cyclin G1 and SUSD2, which contributes to both tumor metastasis and cisplatin resistance of OC (Xu et al., 2018; Xu et al., 2019). As seen in OC, Epstein-Barr virus (EBV)-associated nasopharyngeal carcinoma and colorectal carcinoma (CRC), inhibition of Notch3 signaling can significantly enhance the cisplatin chemosensitivity of tumor cells, indicating the value of Notch3-targeted therapy (Man et al., 2012; McAuliffe et al., 2012; Tzeng et al., 2014; Xie et al., 2016).

In PTX-resistant OC, the decreased expression of the tumor-suppressive miRNAs miR-136 and miR-150 contributes to the overexpression of Notch3 (Kim et al., 2017b; Jeong et al., 2017). Ectopic expression of miR-136 and miR-150 was found to inhibit Notch3 expression, which suppressed the stemness and angiogenesis of SKpac cells (Kim et al., 2017b; Jeong et al., 2017). In addition, several pre-clinical studies indicated that concomitant treatment with PTX and Notch3-specific inhibitors, including GSI, Small interfering RNA (siRNA) or antibody drugs, can enhance the efficacy of PTX treatment in several tumors, including OC, pancreatic carcinoma (PC), and LC (Groeneweg et al., 2014; Yen et al., 2015; Kang et al., 2016; He et al., 2017; Morgan et al., 2017).

### EGFR-TKIs

Studies have revealed novel mechanisms by which Notch3 induces EGFR-TKI resistance in EGFR-mutated tumors. Notch3 receptor was identified as a substrate for EGFR-mediated tyrosine phosphorylation, and EGFR kinase activity induces tyrosine phosphorylation of Notch3, thus inhibiting Notch3 signaling (Arasada et al., 2014). Unfortunately, EGFR-TKI therapy relieves this inhibition, resulting in Notch3 activation and subsequent CSC enrichment (Arasada et al., 2014). In response to EGFR-TKI therapy of non-small-cell lung carcinoma (NSCLC), Notch3 physically binds to β-catenin in the cytoplasm of tumor cells to activate β-catenin signaling (Arasada et al., 2018). The combination of EGFR-TKIs and a β-catenin inhibitor abrogates the Notch3-dependent activation of β-catenin, which strongly attenuates tumor onset, improving the OS and RFS of NSCLC xenograft mice (Arasada et al., 2018). Zhang et al. found that recovering the expression of miR-150 can directly downregulate Notch3 in TKI-resistant NSCLC cell lines, providing another method for reversing Notch3-mediated TKI resistance (Zhang et al., 2019).

In gliomas and triple-negative BC (TNBC), it was found that Notch3 signaling can promote EGFR expression (Alqudah et al., 2013; Diluvio et al., 2018). Notch3 silencing in TKI-resistant TNBC cells induces EGFR dephosphorylation and promotes its intracellular arrest, which increases tumor cell sensitivity to TKI–gefitinib treatment (Diluvio et al., 2018).

### Gemcitabine

In a clinical study of 71 PC patients, Notch3 was identified as a novel biomarker for predicting the efficacy of gemcitabine (GEM), whereby low Notch3 expression was associated with better GEM treatment efficacy and longer OS of PC patients (Eto et al., 2013). Mechanistically, Notch3 increases the activity of PI3K/Akt signaling in PC cells in response to GEM treatment, and this effect can be reversed by Notch3-specific siRNAs (Yao and Qian, 2010). In addition, Notch3 signaling also contributes to GEM resistance in NSCLC cells. Treatment with GEM and GSI significantly enhances GEM sensitivity and leads to tumor cell apoptosis, but the underlying molecular mechanisms remain unclear (Hu et al., 2018).

## Notch3 in Other Aspects of Cancer Biology

### Notch3 in Cancer Epithelial-Mesenchymal Transition and Metastasis

**FIGURE 6:**
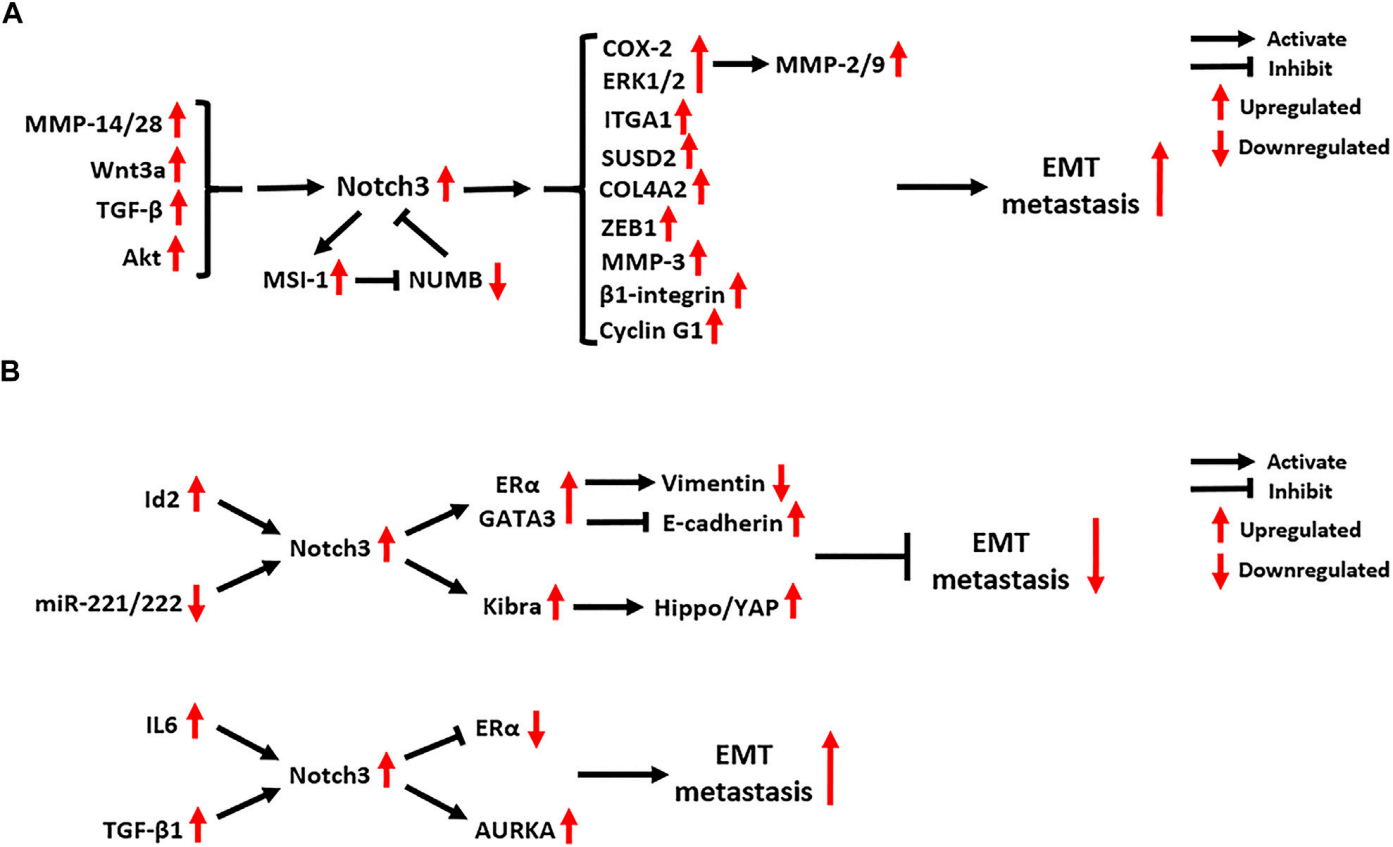
Notch3-related EMT molecular mechanisms in **(A)** tumors other than breast carcinoma and **(B)** breast carcinoma. EMT: Epithelial-Mesenchymal Transition; MMP: Matrix Metalloproteinase; Wnt3a: Wnt Family Member 3A; TGF-β: Transforming Growth Factor Beta; MSI-1: Musashi RNA Binding Protein 1; NUMB: NUMB Endocytic Adaptor Protein; COX-2: Cyclooxygenase 2; ERK1/2: Extracellular-regulated Kinase 1/2; ITGA1: Integrin Subunit Alpha 1; SUSD2: Sushi Domain Containing 2; COL4A2: Collagen Type IV Alpha 2 Chain; ZEB1: Zinc Finger E-Box Binding Homeobox 1; Id2: Inhibitor Of DNA Binding 2; ERα: Estrogen Receptor α; GATA3: GATA Binding Protein 3; IL6: Interleukin 6; KIBRA: KIdney and BRAin; AURKA: Aurora Kinase A.

Notch3 has a close relationship with tumor metastasis ( Figure 6). In clinical studies, high expression of Notch3 was found to be associated with tumor metastasis in OC, NSCLC, prostate carcinoma (PCa), HCC, PC and gallbladder carcinoma (Ye et al., 2013; Zhou et al., 2013; Liu et al., 2016a; Liu et al., 2016b; Zhou et al., 2016; Kim et al., 2017a; Lin et al., 2018; Kim and Gu, 2019). Matrix metalloproteinases (MMPs) cascade with Notch3 signaling and promote tumor metastasis. The Notch3-MMP-3 axis contributes to bone metastasis of PC by promoting the formation of osteoblastic lesions and decreasing osteoblastogenesis (Ganguly et al., 2020). In HCC and pancreatic ductal adenocarcinoma (PDAC), Notch3 signaling activates the COX-2 and ERK1/2 pathways, which subsequently enhance the migration and invasion of tumor cells by upregulating the expression of MMP-2 and MMP-9 (Zhou et al., 2013; Zhou et al., 2016). In addition, MMP-14 and MMP-28 were found to promote tumor metastasis by inducing Notch3 signaling. The MMP-14-Notch3-β1-integrin axis can be activated by interactions between lymphatic endothelial cells and melanoma cells, leading to the transformation of non-metastatic melanoma cells into invasively sprouting melanoma cells (Pekkonen et al., 2018). The MMP-28-Notch3 axis promotes the Epithelial-Mesenchymal Transition (EMT), migration and invasion of HCC cells *in vivo* and *in vitro* (Zhou et al., 2019).

In advanced CRC, Notch3 expression is positively correlated with lymph node as well as distant metastasis, and its expression is dependent on the activation of Akt signaling (Varga et al., 2020). Additionally, DLL4/Notch3 signaling was found to upregulate the expression of the RNA-binding protein MSI-1 in metastatic CRC tumors. Active MSI-1 can inhibit the expression of NUMB, a negative regulator of Notch signaling, which maintains the activation of oncogenic Notch1 and Notch3 signaling pathways (Pastò et al., 2014).

As a key component of Wnt signaling, Wnt3a can activate Notch3 signaling to promote the EMT and metastasis of NSCLC (Li et al., 2015). In bone metastasis of NSCLC, Notch3 signaling is also involved in TGF-β signaling-induced EMT by activating the EMT regulator ZEB1 (Liu et al., 2014a). In metastatic OC tumors, several downstream targets of Notch3 signaling were found to be activated, including type IV collagen (COL4A2), sushi domain containing 2 (SUSD2), Cyclin G1 and integrin subunit alpha 1 (ITGA1) (Brown et al., 2015; Xu et al., 2018; Xu et al., 2019; Price et al., 2020). Blocking Notch3 signaling in metastatic OC can inhibit the adhesion, migration and metastasis of tumor cells, while also enhancing their chemo-sensitivity (Brown et al., 2015; Xu et al., 2018; Xu et al., 2019; Price et al., 2020).

The role of Notch3 in BC metastasis is controversial. A study of 72 BC cases reported that Notch3 expression is correlated with a lower risk of lymph node metastasis, as well as the expression of estrogen receptor α (ERα), progesterone receptor (PR) and GATA3 (Lin et al., 2018). N3ICD in the nucleus of BC cells can bind to the promotors of ERα and GATA3 to promote their expression. ERα and GATA3 activated by Notch3 signaling upregulate vimentin expression and repress E-cadherin expression, which then suppresses the EMT and metastasis of BC by maintaining a luminal phenotype (Dou et al., 2017; Lin et al., 2018). Another Notch3-mediated EMT-suppression mechanism in BC relies on the activation of Hippo/YAP signaling by upregulating the transcription of KIBRA, an upstream factor of Hippo signaling (Zhang et al., 2016b). The inhibitor of DNA binding 2 (Id2), a transcription factor belonging to the bHLH family, can promote the transcription of Notch3, thus attenuating the EMT in BC (Wen et al., 2018). By contrast, microRNAs 221 and 222 were found to target the 3’ UTR of Notch3 and suppress its protein translation in BC cells, which reverses EMT inhibition by Notch3 signaling (Liang et al., 2018).

Although the inhibitory effect of Notch3 on BC metastasis has been confirmed in several studies, a pro-EMT function of Notch3 has been also identified. It was found that the activation of Notch3 signaling is linked to BC seeding and lung/brain metastasis, while abrogation of Notch3 reduces the self-renewal and invasion ability of BC cells, restoring a luminal CD44^low^/CD24^high^/ERα^high^ phenotype (Leontovich et al., 2018). Mechanistically, aberrant Aurora A kinase activity activates Notch3 in breast CSCs and contributes to metastatic growth (Leontovich et al., 2018). In addition, IL6 was found to activate Notch3 signaling in CD133^high^/ERα^low^/IL6^high^ breast CSCs, where it promotes endocrine resistance and metastatic progression (Sansone et al., 2016). In bone-metastatic BC, the activation of JAG1/Notch3 signaling induced by osteoblasts and osteoblast-derived TGF-β1 contributes to aggressive osteolytic metastasis and bone destruction *in vivo* (Zhang et al., 2010). These findings indicate that the relationship between Notch3 and BC metastasis should be explored further.

### Notch3 and Tumor Angiogenesis

**FIGURE 7:**
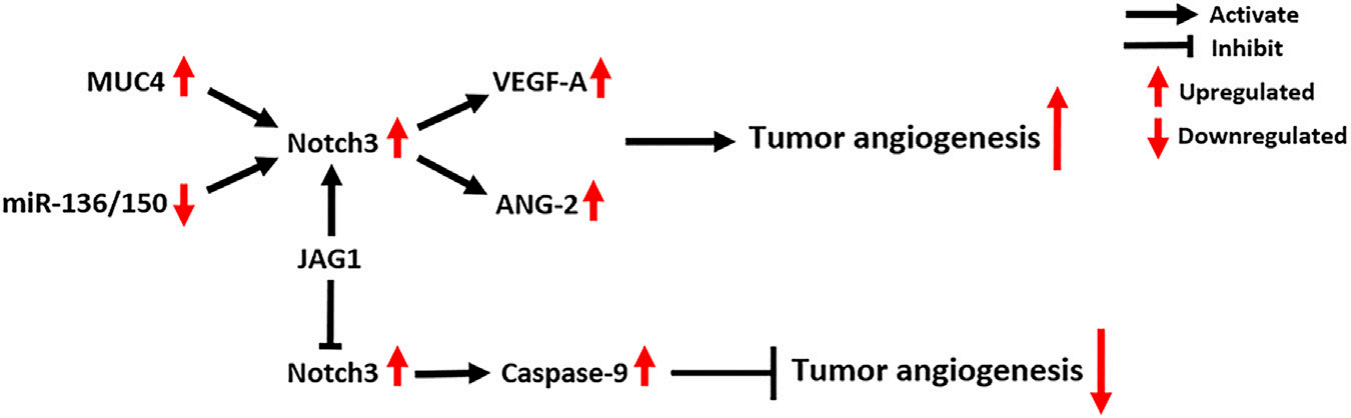
Notch3-related molecular mechanisms in tumor angiogenesis. MUC4: Mucin 4, Cell Surface Associated; VEGF-A: Vascular Endothelial Growth Factor A; ANG-2: Angiogenin 2.

The functions of Notch signaling in tumor vasculature are mainly determined by Notch ligands. JAG1-mediated Notch signaling induces neovascularization and sprouting angiogenesis, while DLL4-mediated Notch signaling inhibits tumor angiogenesis (Xiu et al., 2020b; Xiu et al., 2020a). Notch3 is also involved in the regulation of tumor angiogenesis (Figure 7). Immunohistochemistry for Notch3 expression in 105 TNBC tissues showed that its expression is positively correlated with tumor microvascular density (MVD), which suggests a potential pro-angiogenic role of Notch3 (Xue et al., 2017). MUC4, a large membrane-anchored glycoprotein, can facilitate tumor angiogenesis and increase tumor MVD in PC by activating Notch3 signaling and downstream pro-angiogenic genes, including VEGF-A and ANG-2 (Tang et al., 2016). In addition, Notch3 signaling can be activated by interactions between tumor cells and cells in the tumor microenvironment, which contribute to tumor angiogenesis in several cancers. The interactions between tumor cells and cancer-associated fibroblasts activate Notch3 signaling, thus promoting angiogenesis in oral squamous cell carcinoma (Kayamori et al., 2016). Similarly, the interactions between CSCs and endothelial cells (ECs) activate Notch3 signaling to promote angiogenesis in OC, which can be inhibited by Notch3-targeting miRNAs, including miR-136 and miR-150 (Kim et al., 2017b; Jeong et al., 2017). Additionally, the interactions between CSCs and ECs activate Notch3 signaling to promote angiogenesis and vasculogenic mimicry in melanoma (Hsu et al., 2017).

Notch3 signaling promotes tumor angiogenesis in a canonical CSL-dependent manner, which requires cell-cell interactions and is driven by Notch ligands. However, Notch3 was also found to act as a dependent receptor in tumor ECs to negatively regulate tumor angiogenesis, circumventing CSL (Lin et al., 2017). Mechanistically, Notch3 receptors on the surface of tumor ECs can directly activate caspase-9, which induces the caspase-dependent cell death of ECs (Lin et al., 2017). Interestingly, overexpression of JAG1 ligand can abrogate this effect and promote tumor neovascularization. This indicates that the effects of Notch3 on tumor angiogenesis depend on the cellular context, such as the availability and amounts of Notch ligands in tumors (Lin et al., 2017).

## Notch3-Targeting Strategies for Cancer Therapy

GSIs that prevent the S3 cleavage of Notch receptor are the most commonly used therapeutic option for blocking Notch signaling in cancer (Shih Ie and Wang, 2007). However, they lack specificity and may interfere with the processing of other transmembrane proteins. What’s more, pre-clinical studies have shown that the use of GSIs is associated with severe side effects, such as gastrointestinal tract toxicity (Milano et al., 2004; van Es et al., 2005). Therefore, it is essential to propose other potential methods/strategies that target Notch3 signaling.

### Small Interfering RNAs and Short Hairpin RNAs

siRNAs and shRNAs are powerful molecules that can directly knock down the expression of target genes (Rao et al., 2009). In pre-clinical experiments, Notch3-specific siRNAs or shRNAs were able to prevent Notch3 activation and potently inhibit tumor cell growth *in vivo* and *in vitro* (Yao and Qian, 2010; Serafin et al., 2011; Hassan et al., 2016; Kang et al., 2016; Diluvio et al., 2018). However, the low efficiency of traditional siRNA/shRNA delivery vehicles remains a significant obstacle for their clinical application. To overcome this, researchers developed a novel aptamer-siRNA chimera-based delivery system to inhibit Notch3 expression (Cheng et al., 2017). The chimera consisting of an aptamer and Notch3-specific siRNA was bonded with cationic Au-Fe_3_O_4_ nanoparticles (NPs). This chimera exhibited a high Notch3 silencing efficiency in OC cell lines, as well as potent anti-tumor effects (Cheng et al., 2017). However, there are still no reports on the *in vivo* use of this Au-Fe_3_O_4_ NP-chimera, and its potential value also needs evaluation in clinical studies.

### Non-Coding RNAs

Many non-coding RNAs (ncRNAs), including microRNAs (miRNAs), long noncoding RNAs (lncRNAs) and circular RNAs (circRNAs), play oncogenic or tumor-suppressor roles by regulating Notch3 expression (Table 2) (Song et al., 2009; Furukawa et al., 2013; Liu et al., 2014b; Wang et al., 2015; Cheung et al., 2016a; Zhang et al., 2016a; Kim et al., 2017b; Kim et al., 2017b; Cai et al., 2017; Jeong et al., 2017; Liang et al., 2018; Zhang et al., 2018; Yao et al., 2019; Zhang et al., 2019; Zhu et al., 2019; Cai et al., 2020; Chen et al., 2020; Pei et al., 2020; Zhang et al., 2020; Zong et al., 2020; Kang et al., 2021). MiRNAs are small noncoding nucleotides that directly interact with the 3′-untranslated region (3′-UTR) to degrade a targeted mRNA or inhibit its translation (Vishnoi and Rani, 2017). Most Notch3-targeting miRNAs are tumor-suppressive, and are downregulated in tumor tissues compared to normal tissues. Several lncRNAs and circRNAs were found to “sponge” Notch3-targeting miRNAs, thus resulting in Notch3 overexpression and forming competing endogenous RNA (ceRNA) networks in cancer, including the RUSC1-AS1-miR-7-Notch3 axis (Chen et al., 2020), Circ_PUM1-miR-136-Notch3 axis (Zong et al., 2020), LINC00707-miR-206-Notch3 axis (Zhu et al., 2019), Circ_0058124-miR-218-Notch3 axis (Yao et al., 2019), LINC00210-miR-328-Notch3 axis (Zhang et al., 2018), FAM225A/HOTAIR-miR-613-Notch3 axis (Cai et al., 2017; Zhang et al., 2020), and TUG1-miR-1299-Notch3 axis (Pei et al., 2020). Treatment strategies based on inhibiting the expression of these lncRNAs and circRNAs or recovering the expression of miRNAs have been confirmed to suppress Notch3 expression and block Notch3-controlled oncogenic mechanisms *in vivo* and/or *in vitro* (Song et al., 2009; Furukawa et al., 2013; Liu et al., 2014b; Wang et al., 2015; Cheung et al., 2016a; Zhang et al., 2016a; Cheung et al., 2016b; Kim et al., 2017b; Cai et al., 2017; Jeong et al., 2017; Liang et al., 2018; Zhang et al., 2018; Yao et al., 2019; Zhang et al., 2019; Zhu et al., 2019; Cai et al., 2020; Chen et al., 2020; Pei et al., 2020; Zhang et al., 2020; Zong et al., 2020; Kang et al., 2021).

**TABLE 2:** Non-coding RNAs that target Notch3 in cancer.

Non-coding RNA	Role	Cancer type/cell line	Observations	References
miR-1	Tumor-suppressive	CRC	MiR-1 inhibits *in vitro* tumor cell migration by targeting Notch3	Furukawa et al. (2013)
LncRNA RUSC1-AS1	Oncogenic	HCC	LncRNA RUSC1-AS1 sponges miR-7 to upregulate Notch3 and promotes tumor cell proliferation *in vitro*	Chen et al. (2020)
miR-7	Tumor-suppressive			
Circ_PUM1	Oncogenic	Endometrial carcinoma	Circ_PUM1 sponges miR-136 to upregulate Notch3, and promotes the proliferation, migration and invasion of tumor cells *in vitro* and *in vivo*	Zong et al. (2020)
miR-136	Tumor-suppressive	OC	MiR-136 inhibits the stemness, angiogenesis and chemoresistance of tumor cells *in vitro* by targeting Notch3	Jeong et al. (2017)
miR-150	Tumor-suppressive	OC	MiR-150 inhibits the stemness, angiogenesis and chemoresistance of tumor cells *in vitro* by targeting Notch3	Kim et al. (2017b)
		Lung adenocarcinoma	MiR-150 inhibits tumor cell proliferation *in vitro* by targeting Notch3	Zhang et al. (2019)
miR-96/183	Tumor-suppressive	EBV-associated NPC	MiR-96/183 inhibit stem cell-like properties of tumor cells *in vitro* and inhibit tumor growth *in vivo* by downregulating the expression of NICD3 and NICD4	Cheung et al. (2016b)
miR-206	Tumor-suppressive	HCC, CRC, osteosarcoma and HeLa cells	MiR-206 inhibits the proliferation and migration of tumor cells *in vitro* by targeting Notch3	Song et al. (2009); Liu et al. (2014b); Wang et al. (2015); Cai et al. (2020)
LncRNA 00707	Oncogenic	CRC	LINC00707 sponges miR-206 to upregulate Notch3 and promotes the proliferation and metastasis of tumor cells *in vitro*	Zhu et al. (2019)
Circ_0058124	Oncogenic	Papillary thyroid carcinoma	Circ_0058124 sponges miR-218 to upregulate Notch3, and promotes the proliferation, migration and invasion of tumor cells *in vitro* and *in vivo*	Yao et al. (2019)
miR-218	Tumor-suppressive			
miR-221/222	Oncogenic	BC	MiR-221/222 promote the epithelial-mesenchymal transition of tumor cells *in vitro* by targeting Notch3	Liang et al. (2018)
LncRNA 00210	Oncogenic	NPC	LINC00210 sponges miR-328 to upregulate Notch3 and promotes the proliferation and migration of tumor cells *in vivo* and *in vitro*	Zhang et al. (2018)
miR-328	Tumor-suppressive			
miR-491	Tumor-suppressive	NPC	MiR-491 inhibits the proliferation, migration and invasion of tumor cells *in vitro* and inhibits tumor growth *in vivo* by targeting Notch3	Zhang et al. (2016a)
miR-491/875	Tumor-suppressive	GC	MiR-491/875 inhibit the proliferation, migration and invasion of tumor cells *in vitro* and *in vivo* by targeting Notch3	Kang et al. (2021)
LncRNA FAM225A	Oncogenic	CRC	LncRNA FAM225A sponges miR-613 to upregulate Notch3 and promotes the proliferation, migration and invasion of tumor cells *in vitro*	Zhang et al. (2020)
miR-613	Tumor-suppressive			
LncRNA HOTAIR	Oncogenic	PC	LncRNA HOTAIR sponges miR-613 to upregulate Notch3 and inhibits the proliferation, migration and invasion of tumor cells *in vitro* and *in vivo*	Cai et al. (2017)
LncRNA TUG1	Oncogenic	OC	LncRNA TUG1 sponges miR-1299 to upregulate Notch3 and inhibits tumor cell proliferation *in vitro* and *in vivo*	Pei et al. (2020)
miR-1299	Tumor-suppressive			

Notes: CRC: colorectal carcinoma; HCC: hepatocellular carcinoma; OC: ovarian carcinoma; EBV: Epstein-Barr virus; NPC: nasopharyngeal carcinoma; BC: breast carcinoma; GC: gastric carcinoma; PC: pancreatic carcinoma.

### Antibodies

Antibodies that target Notch receptors/ligands have been confirmed to effectively modulate Notch signaling activity (Xiu et al., 2020b; Gharaibeh et al., 2020). The monoclonal antibodies (mAbs) named A4, A8, MOR20350 and MOR20358, were designed to bind the NRR domain (Lin-Notch repeat (LNR) and heterodimerization domain (HD) domain) of Notch3 protein, which prevents the exposure of the S2 cleavage site and blocks Notch3 activation (Li et al., 2008; Tiyanont et al., 2013; Bernasconi-Elias et al., 2016). In T-ALL harboring Notch3 gain-of-function mutations, anti-Notch3 NRR mAbs show potent anti-leukemic activity in T-ALL cell lines and tumor xenografts (Bernasconi-Elias et al., 2016).

In addition to anti-Notch3 NRR mAbs, another mAb against epidermal growth factor (EGF) repeats of Notch2/3 named tarextumab (also called OMP-59R5) has been used to block Notch2/3 signaling in pre- and clinical studies (O'Reilly et al., 2015; Yen et al., 2015; Hu et al., 2019; Smith et al., 2019). Tarextumab was found to significantly inhibit the growth of PC, BC, OC and small-cell lung carcinoma (SCLC) xenograft tumors, partly by reducing the abundance of CSCs. Additionally, the combination of tarextumab with GEM plus nab-paclitaxel exhibited more potent anti-tumor effects (Yen et al., 2015). In the phase 1b clinical study NCT01647828, tarextumab in combination with gemcitabine plus nab-PTX was evaluated in 38 untreated metastatic PDAC patients, and the overall response rate (ORR) was 29% (O'Reilly et al., 2015). The recommended phase 2 dose was 15  mg/kg with standard doses of the cytotoxic agents. The frequent tarextumab-related emergent adverse events (TEAEs) were diarrhea (60%) and fatigue (43%), which were mostly grade 1 or 2 (O'Reilly et al., 2015). In another phase 1 study (NCT01277146) dose escalation and expansion of tarextumab was evaluated in 42 patients with solid tumors (Smith et al., 2019). Tarextumab was well tolerated at doses of 2.5 mg/kg weekly, as well as 7.5 mg/kg every 14 or 21 days. Diarrhea (81%) was the most common TEAE, followed by fatigue (48%), nausea (45%) and decreased appetite (38%) (Smith et al., 2019). Unfortunately, the results of a phase 2 study (NCT01859741) indicated that tarextumab treatment in combination with platinum-based therapy in 145 untreated SCLC patients did not improve PFS, OS, or ORR of patients (Daniel et al., 2017). In another phase 2 study (NCT01647828) of 177 untreated metastatic PDAC patients, tarextumab treatment in combination with GEM plus nab-paclitaxel also did not improve the OS, PFS, or ORR, while PFS was even statistically worse in tarextumab-treated patients (Hu et al., 2019). Due to the adverse effects of tarextumab shown in phase 2 clinical trials, its clinical development was discontinued.

As mentioned in *EGFR-TKIs*, co-blockage of EGFR and Notch receptors is necessary in some cases. In recent studies, bispecific mAbs targeting both Notch2/3 (tarextumab) and EGFR/HER3 (panitumumab/RG7116/MEHD7945A) have been established using the “Knobs into holes” and “CrossMAb” technologies (Hu et al., 2017; Fu et al., 2019). *In vivo* and *in vitro* experiments on NSCLC and TNBC showed that EGFR/Notch-bispecific mAbs exhibit potent anti-tumor effects, especially decreasing the abundance of CSCs, which limits tumor resistance to EGFR-TKIs and has potential value for clinical applications (Hu et al., 2017; Fu et al., 2019).

### Antibody-Drug Conjugates

Antibody-drug conjugates (ADCs) are mAbs conjugated to small-molecule chemotherapeutic agents via a chemical linker. ADCs can selectively bind to specific targets on the surface of cancer cells and directly deliver the ultra-toxic payload, thus killing cancer cells (Chau et al., 2019). PF06650808, a novel Notch3-targeting ADC, contains a humanized anti-Notch3 IgG1 antibody, a cleavable maleimidocapronic-valinecitruline-p-aminobenzylooxycarbonyl peptide linker, and an auristatin-based cytotoxic payload (Geles et al., 2015). Pre-clinical experiments revealed that PF06650808 can effectively inhibit the growth of TNBC, OC and NSCLC xenograft tumors (Geles et al., 2015). In a recent phase 1, dose-escalation study with 40 solid tumor patients, PF-06650808 was well tolerated at doses ≤2.0 mg/kg, and the maximum tolerated dose was 2.4 mg/kg (Rosen et al., 2020). The most common TEAEs were fatigue (40.0%), decreased appetite (37.5%), nausea (35.0%) and alopecia (32.5%). The ORR and clinical benefit response in the 31 response-evaluable patients was 9.7 and 35.5%, respectively (Rosen et al., 2020). However, the study has been terminated due to a change in sponsor prioritization.

### Histone Deacetylase Inhibitors

Histone deacetylases (HDACs) regulate gene transcription by removing active histone marks such as acetyl groups from ε-N-acetyl lysine on a histone, allowing the histones to wrap the DNA more tightly (Jenke et al., 2021). Several reports indicate that impairing the acetylation/deacetylation balance of Notch3 using HDAC inhibitors (HDACi) that favor hyperacetylation can negatively affect the stability and function of Notch3 in cancer cells and tumor xenograft mouse models (Palermo et al., 2012; Jaskula-Sztul et al., 2015; Zhang et al., 2017; Pinazza et al., 2018). Mechanistically, HDACi such as trichostatin A (TSA), suberoylanilide hydroxamic acid (SAHA) and AB3 increase the ubiquitination and proteasomal/lysosomal degradation of Notch3, which reduces its abundance at the cell surface and impairs Notch3 signaling (Palermo et al., 2012; Jaskula-Sztul et al., 2015; Zhang et al., 2017; Pinazza et al., 2018).

### Other Drugs/Compounds

Temozolomide (TMZ) is an alkylating chemotherapeutic agent that can penetrate the blood-brain barrier and is clinically used in the treatment of glioblastoma (GBM). One study demonstrated that the inhibition of Notch3 expression contributes to TMZ-induced GBM cytotoxicity (Chen et al., 2017). Mechanistically, TMZ enhances the expression of the ER stress protein CHAC1 by activating JNK1/c-JUN signaling. Subsequently, CHAC1 binds to Notch3 protein, which reduces the generation of N3ICD, thus preventing Notch3 signaling (Chen et al., 2017). However, a recent study found that TMZ can also activate DLL4/Notch3 signaling to maintain CSC properties in GBM by upregulating MMP14 expression (Ulasov et al., 2020). Thus, the effect of TMZ on Notch3 signaling needs further exploration.

In HCC chemotherapy, a well-tolerated combination of sorafenib and valproic acid was found to synergistically inhibit tumor growth by downregulating Notch3 and p-Akt (Zhu et al., 2017). Mangiferin, a C-glucosyl xanthone (1,3,6,7-tetrahydroxy-xanthone-C2-β-D-glucoside), can specifically repress Notch3 signaling, which increases apoptosis and inhibits OC tumor growth both *in vitro* and *in vivo* (Zou et al., 2017). In spite of these success stories, more drugs/compounds that potentially target Notch3 should be screened in the future.

## Discussion and Conclusion

Notch3 signaling plays critical roles in cancer progression, and the related molecular mechanisms have been studied in some detail. Stem cell-like properties are a primary feature of Notch3-positive cancer cells, and the overexpression of Notch3 may act as a biomarker for CSCs (See *Notch3 and Cancer Stem Cell Properties*). Notch3 signaling can regulate tumor resistance to chemotherapeutic drugs including doxorubicin, platinum, taxane, EGFR-TKIs, and gemcitabine, which is also dependent on CSCs (See *Notch3 and Drug Resistance*).

To maintain the stemness and proliferation of tumor cells, Notch3 signaling can activate the expression of downstream genes, such as cell cycle-related genes (CCND1, C-MYC and NF-kB1), antiapoptotic genes (SURVIVIN and BCL2), as well as stemness-related genes (OCT-4, ALDH1, NANOG, PBX1, CD44 and CD133) (Park et al., 2010; Man et al., 2012; McAuliffe et al., 2012; Alqudah et al., 2013; Jeong et al., 2017). In addition, there are several associations and cross-talk interactions between Notch3 signaling and other signaling pathways, mainly including the Wnt/β-catenin, Akt, IL6, EGFR, TGF-β and NF-κB signaling pathways, which affect several aspects of cancer cell behavior (Table 3). In tumor metastasis, Notch3 signaling was found to cascade the MMP, Wnt, Akt, IL6 and TGF-β signaling pathways, thereby promoting the invasion and EMT of tumor cells (See *Notch3 in Cancer EMT and Metastasis*). However, anti-EMT properties and mechanisms of Notch3 signaling were also found in BC, suggesting a controversial role of Notch3 in BC metastasis (Zhang et al., 2016b; Dou et al., 2017; Liang et al., 2018; Lin et al., 2018; Wen et al., 2018). Moreover, Notch3 signaling activated by cell-cell interactions between tumor cells and tumor ECs can promote tumor angiogenesis and vasculogenic mimicry (See *Notch3 and Tumor Angiogenesis*). These findings suggest that Notch3 has diverse, complex and wide-ranging roles in tumor cells.

**TABLE 3:** The associations between Notch3 signaling and other signaling pathways.

Pathway	Cancer type	Function	References
Wnt/β-catenin/Notch3	OC	Not shown	Chen et al. (2010)
	NSCLC	Promotes tumor cell cycle progression	Li et al. (2011)
		Promotes tumor cell proliferation and survival	Li et al. (2013)
		Promotes tumor cell invasion and EMT.	Li et al. (2015)
		Promotes tumor cell drug resistance	Arasada et al. (2018)
Notch3/Wnt/β-catenin	HCC	Promotes tumor cell stemness	Zhang et al. (2015)
	BC	—	Papadakos et al. (2019)
Akt/Notch3	CRC	Promotes tumor cell invasion and metastasis	Varga et al. (2020)
	CC	Promotes tumor cell survival	Guest et al. (2016)
Notch3/Akt	BC	Promotes tumor cell stemness	Papadakos et al. (2019)
	GC	Promotes the proliferation, invasion and metastasis of tumor cells	Kang et al. (2021)
	PC	Promotes tumor cell drug resistance	Yao and Qian (2010)
IL6/Notch3	BC	Promotes the stemness, metastasis and drug resistance of tumor cells	Sansone et al. (2016)
Notch3/IL6	BC	Inhibits tumor cell stemness	Wang et al. (2018a)
	NSCLC	Promotes tumor cell EMT and metastasis	Liu et al. (2014a)
EGFR/Notch3	BC	Inhibits tumor cell stemness	Arasada et al. (2014)
Notch3/EGFR	BC	Promotes tumor cell drug resistance	Diluvio et al. (2018)
	Gliomas	Promotes the proliferation, migration and invasion of tumor cells	Alqudah et al. (2013)
TGF-β/Notch3	NSCLC	Promotes tumor cell EMT and metastasis	Liu et al. (2014a)
	BC		Zhang et al. (2010)
Notch3/NF-κB	EBV-associated NPC	Promotes tumor cell proliferation	Man et al. (2012)
	T-cell malignancies	Promotes tumor cell survival	Bellavia et al. (2000); Vacca et al. (2006)
	HCC	—	Qiao et al. (2016)

Notes: OC: ovarian carcinoma; NSCLC: non-small-cell lung carcinoma; EMT: epithelial-mesenchymal transition; HCC: hepatocellular carcinoma; BC: breast carcinoma; CRC: colorectal carcinoma; Akt: AKT Serine/Threonine Kinase; CC: cholangiocarcinoma; GC: gastric carcinoma; PCa: pancreatic carcinoma; IL6: Interleukin 6; EGFR: Epidermal Growth Factor Receptor; TGF-β: Transforming Growth Factor Beta; NF-κB: Nuclear Factor kappa B; EBV: Epstein-Barr virus; NPC: nasopharyngeal carcinoma.

To prevent the abnormal activation of Notch3 signaling in cancer, key Notch3-targeting strategies have been proposed and confirmed effective in pre-clinical studies, including the application of siRNAs/shRNAs, ncRNAs, antibodies, ADCs, and HDACi (See *Notch3-Targeting Strategies for Cancer Therapy*). Different mAbs against Notch3 can specially block Notch3 signaling. However, phase 2 clinical trials of the anti-Notch2/3 mAb drug tarextumab have showed poor efficacy, and the relevant clinical trials have been terminated (Daniel et al., 2017; Hu et al., 2019). Recently, bispecific mAbs targeting both Notch2/3 and EGFR/HER3 have been developed. The main advantage of these bispecific mAbs is that they can target/block both Notch3 and EGFR signaling, which reverses the activation of Notch3 signaling in response to EGFR-TKIs (Hu et al., 2017; Fu et al., 2019). The efficacy of bispecific mAbs may be worth testing in future clinical trials.

ADCs are novel drugs that can kill tumor cells which express specific target molecules. The first Notch3-targeting ADC drug PF06650808 has been evaluated in pre-clinical experiments and a phase 1 clinical trial. Although preliminary, the results demonstrate a manageable safety profile and early signs of anti-tumor activity in cancer patients (Geles et al., 2015; Rosen et al., 2020). Other Notch3-targeting strategies, such as siRNAs/shRNAs, non-coding RNAs and HDACi, have not been tested in clinical trials, so that their efficacy and safety in the treatment of cancer patients need to be evaluated in the future.

In order to develop Notch3-targeting methods/drugs for cancer treatment, the following potential strategies should be considered: 1) Inhibiting Notch3 gene expression using siRNAs, shRNAs, or ncRNAs. 2) Preventing the cleavage of Notch3 protein using small molecules such as using ADAM10 inhibitors (prevent S2 cleavage) and GSIs (prevent S3 cleavage). 3) Antibodies targeting Notch3 protein. 4) Killing Notch3-positive tumor cells by ADCs. 5) Promoting Notch3 degradation by HDACi. Notably, Notch3-specific inhibitors such as antibodies and ADCs are more specific than pan-Notch inhibitors such as GSIs, which may merit further pre- and clinical evaluation.

In summary, Notch3 signaling affects cancer progression through complex molecular mechanisms. Future studies should investigate the relevant mechanisms and exact roles of Notch3 signaling in regulating different cancer behaviors (such as CSC properties, Epithelial-Mesenchymal Transition (EMT), metastasis, drug resistance and angiogenesis) in different tumor types. Furthermore, it is necessary to propose, establish and evaluate more potential Notch3-targeting methods/strategies for cancer treatment.

## Abbreviations

CSL, CBF-1 (RBPJ)/suppressor of hairless/Lag1; TNM, tumor node metastasis; ZIP4, zinc transporter 4; LSD1, lysine-specific demethylase 1; SIRT1, Sirtuin1; akt, AKT serine/threonine kinase; PBX1, PBX homeobox 1; SUSD2, sushi domain containing 2; PI3K, phosphatidylinositol 3-kinase; PD-L1, programmed death ligand 1; mTOR, mammalian target of rapamycin; IL6, interleukin 6; HIF1α, hypoxia inducible factor 1 subunit alpha; COX-2, cyclooxygenase 2; ERK1/2, extracellular-regulated kinase 1/2; MSI-1, musashi RNA binding protein 1; NUMB, NUMB endocytic adaptor protein; TGF-β, transforming growth factor beta; ZEB1, zinc finger E-box binding homeobox 1; GATA3, GATA binding protein 3; KIBRA, kidney and BRAin; NR2F6, nuclear receptor subfamily 2 group F member 6; MUC4, mucin 4, cell surface associated; VEGF-A, vascular endothelial growth factor a; ANG-2, angiogenin 2; CHAC1, cation transport regulator-like protein 1; JNK1, c-jun N-terminal kinase 1; CCND1, cyclin D1; NF-κB, nuclear factor kappa B; OCT-4, organic cation/carnitine transporter 4.
